# Guidelines for Diagnosing and Quantifying Noise-Induced Hearing Loss

**DOI:** 10.1177/23312165221093156

**Published:** 2022-04-26

**Authors:** Brian C. J. Moore, David A. Lowe, Graham Cox

**Affiliations:** 1 Cambridge Hearing Group, Department of Psychology, University of Cambridge, Cambridge, UK; 2 ENT Department, 156705 James Cook University Hospital, Cleveland, UK; 3 ENT Department (retired), 6397 Oxford University Hospitals NHS Foundation Trust, Oxford, UK

**Keywords:** noise exposure, noise-induced hearing loss, diagnosis, quantification, military service

## Abstract

This paper makes recommendations for the diagnosis and quantification of noise-induced hearing loss (NIHL) in a medico-legal context. A distinction is made between NIHL produced by: steady broadband noise, as occurs in some factories; more impulsive factory sounds, such as hammering; noise exposure during military service, which can involve very high peak sound levels; and exposure to very intense tones. It is argued that existing diagnostic methods, which were primarily developed to deal with NIHL produced by steady broadband noise, are not adequate for the diagnosis of NIHL produced by different types of exposures. Furthermore, some existing diagnostic methods are based on now-obsolete standards, and make unrealistic assumptions. Diagnostic methods are proposed for each of the types of noise exposure considered. It is recommended that quantification of NIHL for all types of exposures is based on comparison of the measured hearing threshold levels with the age-associated hearing levels (AAHLs) for a non-noise exposed population, as specified in ISO 7029 (2017), usually using the 50^th^ percentile, but using another percentile if there are good reasons for doing so. When audiograms are available both soon after the end of military service and some time afterwards, the most recent audiogram should be used for diagnosis and quantification, since this reflects any effect of the noise exposure on the subsequent progression of hearing loss. It is recommended that the overall NIHL for each ear be quantified as the average NIHL across the frequencies 1, 2, and 4 kHz.

## Introduction

Despite strict regulations concerning permissible noise exposure in work places, and despite the use of hearing protection, noise-induced hearing loss (NIHL) is still a common problem (Hoffman et al., 2017), especially among workers in mining and construction (Masterson et al., 2015) and among those with military service (Reavis et al., 2021; Swan et al., 2017; Yankaskas, 2013). One reason for this is that hearing protection is not always properly fitted, and even when it is properly fitted it tends to wear out and to fail to provide the stated laboratory values of attenuation in the field (Berger, 2000; Humes et al., 2006; ; Neitzel & Seixas, 2005). Also, for military personnel, hearing protection is not always used, especially during training exercises and during active service when it is necessary to maintain situational awareness.

People who have NIHL produced by noise at work may be eligible for and may claim compensation from their employer. If the employer disputes the claim, then legal proceedings may be instituted to try to enforce the claim. For a claim to be successful, several requirements should be satisfied. Firstly, it should be assessed whether there are plausible causes of hearing loss other than noise exposure. If there are such causes, it should be established that they probably do not fully account for the observed hearing loss. Examples of possible other causes are exposure to ototoxic substances, a family history of hearing loss, and ear infections that have not resolved. Secondly, it should be established that the noise exposure of the individual was sufficient to have the potential for causing a hearing loss. Thirdly, it should be established that the individual has greater hearing loss than would be expected from age alone and also has a pattern of hearing loss indicative of NIHL. In the great majority of cases, this is based solely on the audiogram, even though there is increasing evidence that some of the adverse effects of noise exposure may not be revealed by the audiogram (Billings et al., 2018; Bramhall et al., 2021; Grant et al., 2021; Liberman et al., 2016).

In a medico-legal context, diagnosis of NIHL is based on the “balance of probabilities”, i.e. a diagnosis of NIHL requires a greater than 50% probability of NIHL being present. This is very different from the conventional criterion used in statistical analysis that a certain result should have less than a 5% probability of occurring by chance. The motivation for the present paper stemmed from the experience of the authors that the diagnostic criteria that are commonly employed in the UK, which are discussed in detail below, lead to many people who have a history of noise exposure and who have hearing loss being denied compensation. This applies especially to those who have been exposed to intense impulsive sounds during military service. Given that the diagnostic criteria commonly used in the UK, referred to as the CLB guidelines, were developed over two decades ago and were intended specifically to be appropriate for individuals exposed to steady broadband noise (Coles et al., 2000), it seemed appropriate to re-examine those criteria and to assess whether changes were needed.

If a positive diagnosis of NIHL has been made, then quantification of the amount of NIHL is needed; diagnosis and quantification are two distinct stages of the medico-legal process. The quantification of NIHL requires the effects of age to be partialled out in some way, and there are a number of methods for doing this, which are often based on reference audiograms obtained from a control population with no known noise exposure. It seemed to us that there were also problems with some of the methods that have been used to quantify the amount of NIHL, following a positive diagnosis. Hence this paper also re-examines methods for quantification of NIHL.

It should be noted that in some countries, including the USA, diagnosis of NIHL is usually based on a comparison of audiograms across time, as recommended by the Occupational Safety and Health Administration (1981) and the US Department of Defence (2019). The audiogram obtained at a given time after the noise exposure started is compared with an earlier baseline audiogram. NIHL is deemed to be present when there is “a change in hearing threshold relative to the baseline audiogram of an average of 10 dB or more at 2000, 3000, and 4000 Hz in either ear.” However, this method is based on the assumption that reliable audiograms are obtained at regular intervals, and this is not always the case. In our experience, occupational audiograms are often unreliable, at least in the UK. For example, military veterans have informed us that sometimes they could see when a button was pressed to present a tone, or a light went on when a tone was presented. Sometimes, the tester was reported to nod when a tone was presented. It is not uncommon for occupational audiograms to vary markedly and irregularly across tests taken only a year or two apart. Another problem with the OSHA/DOD method is that noise exposure during military service often results in the greatest hearing loss at 6 and 8 kHz (Lowe & Moore, 2021; Moore, 2020), and this method might fail to diagnose NIHL in such cases. In the present paper, the focus is on methods that are used to diagnose NIHL on the basis of one or more audiograms obtained after noise exposure, where those audiograms have been obtained under known conditions according to a standard method, such as the method recommended by the British Society of Audiology (2018).

In summary, the purpose of this paper is to review methods for the diagnosis and quantification of NIHL and to provide guidelines for the methods that are recommended for assessment, especially in a medico-legal context, where the requirement for a diagnosis is “on the balance of probabilities” rather than with certainty. Note that these are guidelines, not absolute requirements. Each case is different, and there will be some individuals with NIHL who do not meet the requirements for a firm diagnosis. While a positive diagnosis following the guidance provides strong evidence for NIHL, the failure to meet the requirements does not exclude NIHL.

## Medical History

To make a clear diagnosis of NIHL incurred during a specific time period, it is necessary to assess whether there is any other plausible cause of hearing loss, including noise exposure outside the specified time period or outside of the workplace. Of course, it is possible to have NIHL in combination with hearing loss caused in some other way, for example by exposure to jet fuel during military service (Kaufman et al., 2005). The diagnosis of NIHL in such cases is complex, and usually requires the judgment of an otologist, otorhinolaryngologist or ear, nose and throat (ENT) specialist based on a detailed history of the individual case. Often, the effect of the “other” cause of hearing loss can be estimated and allowed for. If the “other” cause, when combined with the effect of age, does not fully account for the observed hearing loss, this makes it likely that NIHL has occurred. However, the focus here is on simpler cases, where causes of hearing loss other than noise exposure and age are excluded as far as possible. For such cases, the following should be excluded:

1. A history of substantial exposure to ototoxic substances, such as solvents (Odkvist et al., 1987);
2. A history of substantial exposure to ototoxic medications, for example during chemotherapy (Baguley & Prayuenyong, 2020);
3. A history of current or previous ear diseases;
4. Head injury associated with auditory symptoms;
5. History of familial hearing loss not caused by noise exposure;
6. Exposure to high levels of noise during leisure activities or outside the time period in question, for example, regular attendance at discotheques, nightclubs or “raves” (Stone et al., 2008).

A conductive hearing loss of 10 dB or more averaged across the frequencies 0.5, 1, 2 and 4 kHz, inferred from the air-bone gap in audiometric thresholds (British Society of Audiology, 2018), does not necessarily rule out the presence of NIHL, but should be noted and taken into account when assessing the audiogram.

The medical history should also include the following information:

1. The types and durations of noise exposures, the sound sources of the exposures and any ear asymmetry in the exposures;
2. The types of hearing protection supplied (if supplied), how well the hearing protection fitted, how often it was replaced, how often it was worn, and whether its use was enforced;
3. Whether and how often periods of temporarily reduced hearing and/or tinnitus were experienced during the time period in question;
4. Whether tinnitus is currently experienced, and when the tinnitus started relative to the period in question. The severity of tinnitus symptoms can be assessed using the guidelines of McCombe et al. (2001) or using a questionnaire such as the Tinnitus Handicap Inventory (Newman et al., 1998), the Tinnitus Functional Index (Meikle et al., 2012), or the Tinnitus Impact Questionnaire (Aazh et al., 2022a).
5. Whether hyperacusis is experienced and if so when the hyperacusis started relative to the period in question. Hyperacusis is an intolerance of sounds that most people do not find to be aversive (Tyler et al., 2014). The severity of hyperacusis symptoms can be assessed using a questionnaire such as the Hyperacusis Questionnaire (Khalfa et al., 2004), the Inventory of Hyperacusis Symptoms (Aazh et al., 2021; Greenberg & Carlos, 2018), or the Hyperacusis Impact Questionnaire (Aazh et al., 2022b).

## Requirement for Sufficient Noise Exposure

A diagnostic method that has been widely used in the UK was proposed by Coles et al. (2000). This method, referred to here as the CLB method, was intended to apply primarily to people exposed to relatively steady broadband noise. The method specifies two requirements in terms of noise exposure, denoted R2(a) and R2(b). R2(a) of the CLB method is that “at least 50% of individuals exposed to this known or estimated amount of noise would be likely to suffer a measurable degree of hearing loss. This noise estimate includes allowance for proper use of hearing protection or for any in-built protection from a conductive hearing loss believed to have been present in the relevant noise-exposure years.” Coles et al. (2000) estimated this requirement to be met when there was “an equivalent daily 8-h continuous noise exposure (L_EP,d_) of not less than 85 dB(A) for a sufficient number of years to lead to a cumulative exposure of at least 100 dB(A) NIL, the so-termed Noise Immission Level.”

This requirement seems to us to be excessively stringent. If a given NIL is sufficient to produce NIHL in 50% of individuals, then it follows that at least some individuals would experience NIHL for lower exposure levels. Fairness to a claimant requires only that the noise exposure should be sufficient to produce NIHL in a reasonable proportion of individuals. This problem was acknowledged by Coles et al. (2000), and led them to introduce CLB requirement R2(b): “Substantial amounts of NIHL can be caused in a minority of persons exposed to < 100 dB(A) NIL; that is, in those who are more than averagely susceptible. To allow for such cases, a less stringent noise exposure requirement is applicable provided the audiometric evidence of noise damage is stronger. The lower level of total noise exposure for such cases is reduced to 90 dB(A) NIL, although the lower limit on L_EP,d_ remains at 85 dB(A)”. The CLB guidelines suggest that R2(b) should be applied when there is a notch or bulge in the audiogram whose depth meets requirement R3(b); this is described later in this paper.

A problem with R2(a) is that an NIL of 100 dB(A) is probably higher than the NIL required for 50% of individuals to experience NIHL. Passchier-Vermeer (1974) presented evidence showing that exposure to steady noise with a noise rating (NR) of 85 dB [approximately equal to 90 dB(A)] for eight hours per day for five days per week for ten years, giving an NIL of approximately 100 dB(A), is sufficient to produce a median hearing loss of 17 dB at 4 kHz. This indicates that a criterion NIL of 100 dB(A) is higher than appropriate. She showed further that a 10-dB lower exposure [a NR of 75, equivalent to 80 dB(A) for the same duration, giving an NIL of approximately 90 dB(A)] led to a hearing loss of 11 dB at 4 kHz for the 10^th^ percentile, i.e. that lower NIL had the potential to produce hearing loss in some individuals. In our opinion, a criterion NIL of 90 dB(A) is appropriate, since this will lead to NIHL in a small proportion of individuals. We recommend an NIL of 90 dB(A), with no lower limit on L_EP,d_, in all cases of exposure to broadband steady noise.

Additional considerations arise when the individual has been exposed to impulsive sounds, for example from hammering or gunshots. It is well established that, for a given root-mean square exposure, impulsive sounds are more damaging to the auditory system than steady sounds (which usually have a Gaussian distribution of instantaneous amplitudes) (Henderson & Hamernik, 1986; Shi et al., 2021). In a systematic review, Shi et al. (2021) concluded that “The A-weighted equivalent continuous sound pressure level (LAeq) is not a sufficient measurement metric for quantifying non-Gaussian noise exposure, and a combination of kurtosis and noise energy metrics (e.g., LAeq) should be used. It is necessary to reduce the exposure of non-Gaussian noise to protect the hearing health of workers.” Unfortunately, there is at present no consensus as to what the appropriate combination measure should be. Shi et al. (2021) showed that the prevalence of high-frequency NIHL (HFNIHL, defined as a hearing threshold level ≥25 dB HL, averaged across 3, 4 and 6 kHz) was 33.3% for workers exposed to non-Gaussian noise as opposed to 27.7% for workers exposed to Gaussian noise of the same cumulative level. This change in prevalence of 5.6% is about 0.78 times the increase in prevalence of 7.2% produced by changing the cumulative noise exposure from 85 to 90 dB(A); see Table 5 of Shi et al. (2021). Hence, exposure to non-Gaussian noise, on average, has an effect similar to increasing the exposure level by about 4 dB (5 × 0.78). Hence, for cases of exposure to non-Gaussian noise, it would seem reasonable at present to use a limit of 86 dB(A) NIL, i.e. 4 dB lower than for exposure to steady broadband noise.

Hearing loss sustained during military service, denoted here M-NIHL, is a special case. It can involve exposure to peak sound levels exceeding 150 dB SPL (Jokel et al., 2019), which are capable of damaging the ear immediately when hearing protection is not worn, is of insufficient effectiveness, or is inadequately fitted. To accrue a NIL of 100 dB(A) to satisfy requirement R2(a) of the CLB guidelines would require the firing of 160 rounds per shift, unprotected, for five days per week for approximately seven years, giving a total of more than 250,000 rounds. Even the lower R2(b) requirement of the CLB guidelines would require unprotected exposure to 25,000 rounds. In fact, it has been shown that a relatively small amount of unprotected exposure (100 rounds or less) when practicing the shooting of rifles can produced significant hearing loss (Keim, 1969; Moon et al., 2011). Hence, both R2(a) and R2(b) of the CLB guidelines are clearly inapplicable in the case of exposure to intense impulsive sounds, as was acknowledged by the authors of the CLB guidelines (Coles et al., 2000).

In the great majority of cases, it is impossible to quantify precisely the noise exposure of a specific individual during military service. However, it is likely that all military personnel who have seen active service have been exposed to potentially damaging sounds. Consistent with this, Jokel et al. (2019) stated, “All military personnel are going to be exposed to loud sounds. In fact, they are likely to have exposure to some of the most intense sounds that can be found in any occupation.” Evidence that military noise exposure is typically sufficient to cause hearing loss in a substantial proportion of men is provided by Figure 1 in Moore (2020), showing that about 50% of professional military personnel have hearing loss in the frequency range 6–8 kHz, and by Figure 2 in Moore (2020), showing that the mean hearing loss after 10 years of military service is greater than 30 dB at 4, 6, and 8 kHz. Also, in our experience it is near universal that those claiming compensation for M-NIHL report times where their hearing was dulled and/or where they experienced temporary tinnitus. Such reports are generally accepted as indicating potentially or actually damaging noise exposure (Brungart et al., 2019; Kryter, 1963).

Another special case is for individuals exposed to intense tones rather than noises. Such exposures can result from the use of “Tone Set Equipment” (TSE), which has been used in the past to test the integrity of telephone lines. The sounds produced by TSE are typically 1-kHz tones with levels up to 137 dB SPL. Exposures of this type are sometimes described as producing “acoustic shock”, and they can lead to immediate hearing loss as well as tinnitus, hyperacusis, and psychological effects (Davis et al., 1950; McFerran & Baguley, 2007; Westcott, 2006). While “safe” exposure limits for tones have not been established, it can reasonably be assumed that exposure to tones with levels over 130 dB SPL is likely to have the potential for damaging the ear, and that all people who have worked with TSEs have had potentially damaging exposures. We denote this type of hearing loss as T-NIHL.

In summary, for people who have been exposed primarily to steady broadband noise, we recommend that a total noise exposure of 90 dB(A) NIL is taken as sufficient to have the potential for causing NIHL. For people who have regularly been exposed to impulsive sounds in non-military occupations, a lower limit of 86 dB(A) NIL should be used. It can reasonably be assumed that all those who have seen active military service or who have worked with TSE have been exposed to sounds with the potential to cause hearing loss.

## Diagnosis Based on Audiometric Configuration

### Cases of Exposure to Steady Broadband Noise

Several methods for diagnosing NIHL are based on the typical shapes of the audiograms produced by exposure to steady intense broadband noise. Such audiograms typically show a notch or downward bulge in the audiogram in the frequency region 3–6 kHz (Passchier-Vermeer, 1974; Smoorenburg, 1992). The reasons for this are as follows:

1. The ear canal produces an acoustic resonance that boosts the sound level at the eardrum (relative to that measured with a microphone placed at the centre of the position of the listener's head, when the listener is removed from the sound field) by about 15 dB for frequencies close to 3 kHz (Moore, 2012; Shaw, 1974). Hence, for a typical broadband sound, the level at the eardrum is greater for frequencies close to 3 kHz than for lower or higher frequencies. The centre frequency of the ear canal resonance depends on the geometry of the ear canal and varies across individuals from about 2 to 4 kHz.
2. Each place on the basilar membrane within the cochlea is tuned to a certain frequency, called the characteristic frequency (CF) (Moore, 2012). However, the CF depends on sound level (McFadden, 1986; Moore et al., 2002). The place on the basilar membrane with a CF of 4 kHz at low and medium sound levels responds most strongly to frequencies close to 3 kHz at very high sound levels.

Because of these two effects, exposure to an intense broadband noise produces maximum damage to the hair cells in the cochlea at a place whose CF is close to 4 kHz, and it is this damage that is measured in the audiogram.

The existence of a “noise notch” or calculated bulge provides the basis for several diagnostic methods (Coles et al., 2000; Niskar et al., 2001; Phillips et al., 2010), all of which depend on the hearing threshold levels (HTLs) at 3, 4 or 6 kHz being higher (worse) than the HTLs at lower frequencies (e.g. 1 kHz) and higher frequencies (e.g. 8 kHz). For a description of these methods, see Lowe and Moore (2021). As an illustration of these methods, we describe here the audiometric requirements of the CLB method, which is the most widely used method in the UK in medico-legal cases. The CLB method includes a recommendation that HTLs at 6 kHz should be “adjusted” (reduced) by 6 dB when Telephonics TDH39 headphones are used, to allow for a “calibration artefact” that depends on the coupler used for calibration (Lawton, 2005). However, the coupler that is used in most countries does not lead to an artefact, and recent evidence suggests that such an adjustment is not appropriate in the UK (Lowe & Moore, 2021). Therefore, we recommend that no adjustment is made. The requirements of the CLB method are:

R1. A single measurement of the HTL at 3, 4 or 6 kHz should be at least 10 dB greater than the HTL at 1 or 2 kHz.
R2(a) and R2(b) are the requirements for sufficient noise exposure, as described earlier.
R3(a). This requirement applies when R2(a) is met. There should be a downward notch or bulge in the audiogram in the range 3–6 kHz. A notch is defined as present when “the HTL at 3 and/or 4 and/or 6 kHz …. is at least 10 dB greater than at 1 or 2 kHz and at 6 or 8 kHz”. A bulge is defined as present when “the HTL at 3 and/or 4 and/or 6 kHz … is at least 10 dB greater relative to the comparison values for age-related hearing loss at corresponding frequencies.” To establish whether R3(a) is satisfied, a “bulge analysis” is conducted using the HTLs at 1 or 2 kHz and at 6 or 8 kHz as “anchor points”. R3(a) is based on the assumption that NIHL will typically result in greater hearing loss at 4 kHz than at 1 or 2 and 6 or 8 kHz.
R3(b). This requirement applies when R2(a) is not met, but R2(b) is met. R3(b) is similar to R3(a), except that the notch or bulge in the audiogram must have a value of 20 dB or more, instead of 10 dB or more.

In the literature, the term percentile has been used in different ways. Sometimes a low percentile has been taken to correspond to poorer than typical hearing (ISO 7029, 2017), while sometimes a low percentile has been taken to correspond to better than typical hearing (Flamme et al., 2020). In what follows, we adopt the convention that a lower percentile corresponds to poorer than typical hearing. For example, for a given age, 75% of individuals would have better hearing than the 25^th^ percentile.

An example of a bulge analysis using the CLB method, for a man exposed to broadband steady noise in a factory, is shown in Table 1. R2(a) was satisfied. For this example, the anchor points were taken as 1 and 8 kHz, which are the most commonly used anchor points. The age-associated hearing loss (AAHL) values are those for a man without noise exposure aged 50 years at the 25^th^ (worst) percentile, taken from Table 2 of Coles et al. (2000). The percentile is chosen to match the HTLs at the chosen anchor points as closely as possible. The measured HTL at 1 kHz is 3 dB higher than the AAHL value, while the HTL at 8 kHz is 6 dB lower than the AAHL value. These are denoted “misfit values”. They indicate the extent to which the AAHL values at the anchor points differ from the measured HTLs. Note that Tables 2 (for men) and 3 (for women) of Coles et al. (2000) give AAHL values based on a now-obsolete standard (ISO 7029, 1984) that was adjusted (to give higher HTLs) based on the data presented in Lutman and Davis (1994). Also, the tables give AAHL values only for the 25^th^, 50^th^ and 75^th^ percentiles and only for ages up to 70 years at five-year intervals, so the values are quite coarsely quantized. The misfit values are interpolated across frequency on a logarithmic frequency scale (line D) and used to give adjusted AAHL values (the sum of rows C and D). These adjusted AAHL values are set equal to the measured HTL when they are greater (worse) than the measured HTL, since noise exposure is generally accepted not to improve HTLs. The differences between the adjusted AAHL values and the measured HTLs are shown in the bottom line of the table; these correspond to the estimated NIHL. Any value exceeding 10 dB at 3, 4, or 6 kHz qualifies as a bulge. In this case, R3(a) is not satisfied; the largest estimated NIHL is 8 dB at 4 kHz.

**Table 1.** Example of a Bulge Analysis Using the CLB Method.

	Frequency, kHz	1	2	3	4	6	8
A	Hearing threshold level (HTL), dB HL	15	10	15	40	40	40
B	HTL at selected anchor points	15					40
C	Selected age-associated hearing loss (AAHL)	12	19	25	35	39	46
	Misfit values (dB) = B − C at anchor points	3					−6
D	Interpolated misfit values (dB)	3.0	0.0	−1.5	−3.0	−4.5	−6.0
	Adjusted AAHL = C + D	15.0	19.0	23.5	32.0	34.5	40.0
	Set AAHL to 0 when AAHL<0	15.0	19.0	23.5	32.0	34.5	40.0
E	Set AAHL to actual when AAHL>actual	15.0	10.0	15.0	32.0	34.5	40.0
	NIHL, i.e. bulge (dB) = A–E, rounded	0	0	0	8	6	0

Values in specific lines are denoted A, B, C, D, and E. The AAHL values are those for a man aged 50 years at the 25^th^ (worst) percentile, as given in table 2 of Coles et al. (2000).

It should be noted that although the CLB diagnostic method is currently the most widely used method in the UK, there have not, to our knowledge, been any published studies of its sensitivity in diagnosing NIHL produced by exposure to steady broadband noise. One reason for this is that there is no generally accepted “gold standard” for deciding whether or not a diagnosis of NIHL is correct. The specificity of the method (the percentage of people without NIHL who are diagnosed as not having NIHL) was estimated for a non-noise-exposed control population by Moore and von Gablenz (2021) to be 87% when each ear was considered separately.

Relatively recently, an updated ISO standard has been published based on populations that were carefully screened to exclude individuals with conductive hearing loss or noise exposure (ISO 7029, 2017). The Introduction in ISO 7029 (2017) includes the statement: “Hearing thresholds presented in this document are generally lower at high frequencies than those in the previous editions of this document. The 4 kHz dip observed in males has become negligibly small. The source data of the previous editions might not have been screened rigorously in terms of hearing abnormalities. Problems related to instrumentation might also have affected measurement data”. The section headed “Scope” in ISO 7029 (2017) includes the statement: “The data are applicable for estimating the amount of hearing loss caused by a specific agent in a population. Such a comparison is valid if the population under study consists of persons who are otologically normal except for the effect of the specific agent. Noise exposure is an example of a specific agent”. These two statements provide good reasons for not using earlier versions of the standard and for not using the tabulated values in Coles et al. (2000), which in any case contain several erroneous entries.

The equations given in ISO 7029 (2017) can be used to calculate AAHL values for any desired age (up to 80 years) and percentile. This can sometimes change the outcome of the bulge analysis. Table 2 shows a bulge analysis based on the same case as for Table 1, but using AAHL values taken from ISO 7029 (2017) for a man aged 50 years at the 9^th^ percentile. The measured HTL at 1 kHz is 1.9 dB higher than the AAHL value, while the HTL at 8 kHz is 1.4 dB lower than the AAHL value. When the AAHL values from ISO 7029 (2017) are used, the R3(a) CLB requirement is met; the estimated NIHL at 4 kHz is 11 dB.

**Table 2.** As Table 1 but using AAHL values for a man aged 50 years at the 9^th^ (worst) percentile, as given in ISO 7029 (2017).

	Frequency, kHz	1	2	3	4	6	8
A	Hearing threshold level (HTL), dB HL	15	10	15	40	40	40
B	HTL at selected anchor points	15					40
C	Selected age-associated hearing loss (AAHL)	13.1	19.5	24.5	29.0	36.0	41.4
	Misfit values (dB) = B − C at anchor points	1.9					−1.4
D	Interpolated misfit values (dB)	1.9	0.8	0.2	−0.3	−0.8	−1.4
	Adjusted AAHL = C + D	15.0	20.3	24.8	28.7	35.1	40.0
	Set AAHL to 0 when AAHL<0	15.0	20.3	24.8	28.7	35.1	40.0
E	Set AAHL to actual when AAHL>actual	15.0	10.0	15.0	28.7	35.1	40.0
	NIHL, i.e. bulge (dB) = A–E, rounded	0	0	0	11	5	0

It should be noted, as acknowledged by Coles et al. (2000), that the NIHL estimated using the CLB method for diagnosis underestimates the true extent of the NIHL, because the noise exposure often affects the HTLs at the anchor points (Passchier-Vermeer, 1974; Smoorenburg, 1992). Hence, as stated by the authors, the CLB method should not be used to quantify NIHL.

For cases of exposure to broadband steady noise, we recommend use of a modified version of the CLB method. The requirements of the modified version, denoted (mod), are as follows:

R1(mod). A single measurement of the HTL at 3, 4 or 6 kHz should be at least 10 dB greater than the HTL at 1 kHz or 2 kHz. This is actually the same as R1.
R2(mod). There should be evidence for an NIL of 90 dB(A) or more. The reasons for this lower NIL were given earlier in this paper.
R3(mod). There should be a downward notch or bulge in the audiogram in the range 3–6 kHz. A notch is defined as present when the HTL at 3 and/or 4 and/or 6 kHz is at least 10 dB greater than at 1 and 8 kHz. A bulge is defined as present when the HTL at 3 and/or 4 and/or 6 kHz is at least 10 dB greater than expected from AAHL values. To establish whether R3(mod) is satisfied, a bulge analysis using the HTLs at 1 kHz and at 8 kHz as “anchor points” should be conducted, as illustrated in Table 2. The AAHL values should be based on ISO 7029 (2017). The percentile should be chosen to minimize the mismatch between the measured HTLs and the AAHL values at the anchor points of 1 and 8 kHz, taking into account the sign of the mismatch (for example, a mismatch of −4 dB at 1 kHz and + 4 dB at 8 kHz would reflect the correct choice of percentile, while a mismatch of −4 dB at both 1 and 8 kHz would indicate the need to choose a different percentile). In some cases, it may be appropriate to change the lower anchor frequency to 2 kHz and/or the upper anchor frequency to 6 kHz when the HTLs at 1 and/or 8 kHz are “out of line” with those at other frequencies. The lower anchor frequency should be changed to 2 kHz when the HTL at 2 kHz is 10 dB or more better than the HTL at 1 kHz and the upper anchor frequency should be changed to 6 kHz when the HTL at 6 kHz is 10 dB or more better than the HTL at 8 kHz.

### Cases of Exposure to Impulsive Sounds in Industry

For cases of exposure to impulsive sounds in industrial settings, we recommend that diagnosis is based on the modified CLB method described above, except that R2(mod) is changed to: there should be evidence for an NIL of 86 dB(A) or more.

### Cases of Exposure to Intense Impulsive Sounds

In this section, we consider cases of exposure that include very intense impulsive sounds, such as can occur in military service. The exposure may also include more steady sounds, such as the noise of jet engines or the interior of tanks. Noise exposure during military service typically leads to hearing losses that are greatest at 4, 6 and 8 kHz, and the mean loss at 8 kHz is similar to or greater than that at 4 kHz (Attias et al., 2004; Humes et al., 2006; Lowe & Moore, 2021; Moore, 2020; Walden et al., 1975; Ylikoski & Ylikoski, 1994). For some individuals, M-NIHL is greater at 8 kHz than at lower frequencies (Lowe & Moore, 2021; Moore, 2020). Also, the HTL for frequencies as low as 0.5 and 1 kHz can be markedly affected by noise exposure during military service (Lowe & Moore, 2021). For these reasons, methods for diagnosing NIHL based on the assumption that HTLs are most affected for frequencies close to 4 kHz and are relatively unaffected for frequencies of 1 and 8 kHz are not appropriate for diagnosing M-NIHL.

To illustrate this, Lowe and Moore (2021) estimated the sensitivity (the percentage of cases with NIHL correctly diagnosed as having NIHL) of three methods for diagnosing NIHL based on identification of a notch or bulge in the audiogram when applied to a sample of 80 men with a high probability of having M-NIHL (it is relatively rare for women to make claims for M-NIHL). All of the men were claiming compensation for M-NIHL. All reported exposure to intense impulsive sounds produced by rifles, machine guns, grenades, shoulder-mounted anti-tank weapons, thunder flashes, and mortars, sometimes without hearing protection. Nearly all of the sample reported times when they had a temporary dulling of hearing (also known as temporary threshold shift) and/or tinnitus following such exposure. One of the methods was the CLB method described earlier. The other methods were those of Niskar et al. (2001) and Phillips et al. (2010), which have been used for epidemiological studies in the USA. The highest overall sensitivity of 72.5% was for the method of Phillips et al. (2010). The Niskar method gave a sensitivity of only 27%, largely because of their requirement that for a positive diagnosis the HTLs at 0.5 and 1 kHz should be ≤ 15 dB HL, while the results of Lowe and Moore (2021) suggest that HTLs at these frequencies can be affected by M-NIHL. The CLB method gave a sensitivity of 70%. For the CLB and Niskar methods, negative diagnoses occurred mainly when the HTL at 8 kHz was equal to or greater than the HTL over the frequency range 3–6 kHz.

The reasons why noise exposure during military service produces very variable audiometric outcomes are not clear. However, the high variability is consistent with the high variability in the patterns of hearing loss found in animals that have been exposed to intense impulsive sounds (Henderson & Hamernik, 1986). It may be the case that intense impulsive sounds produce strong excitation over a large proportion of the basilar membrane within the cochlea, and that the basal region, which responds best to high frequencies, is more susceptible to damage than more apical regions (Robles & Ruggero, 2001). The high variability may also be related to the variety of the spectral shapes of the sounds encountered in military service (Jokel et al., 2019).

Moore (2020) proposed a new method for the diagnosis of M-NIHL, based on the patterns of the audiograms that are typically found following noise exposure during military service. The characteristics of M-NIHL are often similar to those of age-related hearing loss, also called presbycusis (with the exception that presbycusis usually involves similar hearing loss for the two ears, while M-NIHL is often markedly asymmetric, Lowe & Moore, 2021). This makes a definite diagnosis of M-NIHL difficult for some individuals aged over about 40 years. However, in some (but not all) cases it is possible to distinguish M-NIHL from presbycusis, based on the observation that in cases of presbycusis the hearing loss is typically greater at 8 kHz than at 3, 4 or 6 kHz. For a man at the 50^th^ percentile who has not experienced significant noise exposure, the difference between the HTLs at 8 and 6 kHz is about 1 dB at age 40 years, increasing to about 9 dB at age 70 years (ISO 7029, 2017). Similarly, the difference between the HTLs at 8 and 4 kHz is about 2 dB at age 40 years, increasing to about 17 dB at age 70 years and the difference between the HTLs at 8 and 3 kHz is about 3 dB at age 40 years, increasing to about 23 dB at age 70 years. In contrast, as described above, M-NIHL is on average greater at 6 than at 8 kHz and is on average similar at 4 and 8 kHz. Also, the maximum hearing loss sometimes falls at 3 kHz. Hence, a diagnosis of M-NIHL can be made with good confidence if the following requirements are satisfied (M here denotes the method of Moore):

R1M. A single value of the HTL at 3, 4, 6, or 8 kHz is at least 10 dB higher than the HTL at 1 or 2 kHz. This is similar to requirement R1 of the CLB method, except that the frequency of 8 kHz has been added to allow for the fact that noise exposure during military service typically produces the greatest hearing losses at 4, 6, and 8 kHz, but sometimes produces the greatest loss at 3 kHz.
R2aM. The difference between HTLs at 8 and 6 kHz is at least 5 dB smaller than would be expected from age alone or the difference between HTLs at 8 and 4 kHz or between 8 and 3 kHz is at least 10 dB smaller than would be expected from age alone, based on the median values in ISO 7029 (2017). For example, at 4 kHz R2aM is satisfied if
(1) [HTL(8)−HTL(4)+10]≤[AAHL(8)−AAHL(4)],
  where HTL(*x*) is the HTL at frequency *x* (kHz) and AAHL(*x*) is the AAHL at frequency *x* (kHz). This is similar to the methods based on identifying a notch or bulge in the audiogram, but is based on the fact that noise exposure during military service typically leads to less hearing loss at 8 than at 6 kHz, and to similar hearing loss at 4 and 8 kHz, and sometimes leads to the greatest hearing loss at 3 kHz, whereas age alone typically leads to greater hearing loss at 8 than at 3, 4 or 6 kHz.

If requirements R1M and R2aM are met, this provides reasonably strong evidence for M-NIHL, since the shape of an audiogram required to meet R2aM is different from the shape associated with age alone. If requirement R2aM is not met, this does not imply the absence of M-NIHL, since noise exposure during military service can have a substantial effect, and sometimes its maximal effect, on the HTL at 8 kHz. If requirement R2aM is not met, then a diagnosis of M-NIHL can be made if R1M is met, and the following requirement is met:

R2bM. The HTL at any one of 4, 6, or 8 kHz is at least 20 dB higher than the median threshold for each frequency expected for that age, based on ISO 7029 (2017). The frequencies of 4, 6, and 8 kHz were chosen because these are the frequencies that are usually most affected by noise exposure during military service, but the exact frequency showing the greatest loss varies across individuals (Lowe & Moore, 2021; Moore, 2020). The value of 20 dB was chosen for several reasons: (1) To avoid a high number of false-positive diagnoses; (2) Because 20 dB is greater than the typical errors associated with measurement of an audiogram (Margolis et al., 2010); (3) Because a 20-dB threshold elevation at high frequencies is likely to lead to a measurable reduction of the ability to understand speech in noise (Smoorenburg, 1992).

In summary, for the method of Moore (2020), R1M must be met and either R2aM or R2bM or both must be met.

Two important characteristics of any diagnostic test are its sensitivity and its specificity (the percentage of people without M-NIHL who are diagnosed as not having M-NIHL). The specificity of the diagnostic method of Moore (2020) was assessed by Moore and von Gablenz (2021), using a sample of 1903 adults, mostly based in two medium-sized cities in the northwest of Germany. The sample was initially restricted to males aged between 29 and 60 years [the same as for the noise-exposed sampled assessed by Moore (2020)]. The sample was then further screened to match their characteristics to those of the noise-exposed sample, except for the noise exposure.

When applied to the sample of 58 military veterans studied by Moore (2020), Moore's method was found to have an overall sensitivity of 96.5%. When applied to the independent sample of 80 military veterans studied by Lowe and Moore (2021), the method was found to have an overall sensitivity of 95%. These sensitivity values are very high and markedly greater than for the methods of Coles et al. (2000), Niskar et al. (2001) and Phillips et al. (2010). For the standard combination of requirements [R1M, and (R2aM or R2bM)] the specificity of Moore's method was 67%, which is only moderate. For R1M and R2aM alone, the specificity was 86%. For R1M and R2bM alone, the specificity was 76%. For R1M and both R2aM and R2bM, the specificity was 94%. Thus, the specificity was greater when all three requirements were met than when only R1M and R2aM or R1M and R2bM were met.

A measure of the performance of a diagnostic method can be derived from the proportion of “hits” (sensitivity) and “false alarms” (1 − specificity):

(2) $$d{}^{\prime }=Z(\mathrm{hit}\;\mathrm{rate})\text{–}Z(\mathrm{false}\;\mathrm{alarm}\;\mathrm{rate}),$$

where function *Z*(*p*), *p* ∈ [0,1], is the inverse of the cumulative Gaussian distribution (Green & Swets, 1974). The higher the value of *d′*, the better is the performance of the method. For the method of Moore (2020), and for each ear considered separately, the value of *d′* for the standard combination of requirements was 2.3, which is conventionally considered as reasonably high. For comparison, *d′* was estimated for the CLB method using anchor points of 1 and 8 kHz. When applied to each ear separately, the CLB method gave a sensitivity of 0.69 and a false-positive rate of 0.13, leading to a *d′* value of 1.6, markedly lower than for the method of Moore (2020).

We conclude that for cases of noise exposure during military service, the method of Moore (2020) is preferable to methods based on the identification of a notch or bulge in the audiogram centered near 4 kHz. Confidence in a positive diagnosis is greatest when R1M, R2aM and R2bM are all met, since specificity is greatest in that case, at 94%. Confidence is somewhat lower, but still high, when only R1M and R2aM are met, since R2aM requires an audiogram shape different from that produced by age alone, and since specificity is still reasonably high, at 86%. Confidence is lower (but still with a probability greater than 50%) when only R1M and R2bM are met, which is associated with a specificity of 76%.

Confidence in a positive diagnosis is greater when the outcome is positive for both ears as opposed to only one ear. However, M-NIHL is often asymmetric across the two ears, and the asymmetry in HTLs can often be associated with asymmetric exposure (Keim, 1969; Lowe & Moore, 2021). Hence, asymmetry in the HTLs across ears can be taken as supporting the presence of M-NIHL (Lowe & Moore, 2021). Because of this asymmetry, the diagnosis can sometimes be positive for one ear, but not for the other ear. However, M-NIHL can sometimes be symmetric across the two ears, so a lack of asymmetry does not imply the absence of M-NIHL.

Exposure to broadband noises in industrial situations when the noise level is unusually high or the exposure duration is very long can lead to NIHL that spread towards higher frequencies, including 6 and 8 kHz (Passchier-Vermeer, 1974). In such cases, there may be no notch or bulge in the audiogram, and diagnostic methods that depend on the presence of a notch or bulge will fail. The method of Moore (2020), while originally intended for the diagnosis of M-NIHL, may also be applied in such cases. We recommend that the method of Moore (2020) be applied in preference to the modified CLB method in cases when the NIL is 100 dB(A) or more, since such exposure often leads to marked NIHL at 6 and 8 kHz (Passchier-Vermeer, 1974).

### Cases of Exposure to Intense Tones

Very intense tones presented via headphones, as is the case with TSE, produce a distribution of stimulation along the basilar membrane within the cochlea that is very broad. Places with a wide range of CFs at and above the frequency of the exposure tone are stimulated with a high intensity. However, for structural and metabolic reasons, the places with high CFs are most vulnerable to damage (Borg et al., 1995). Hence, the maximum damage caused by exposure to intense tones is likely to occur for frequencies above that of the exposure tone. However, there is no reason to expect the maximum T-NIHL to occur for frequencies close to 4 kHz. Data on permanent hearing loss caused by exposure to intense tones are sparse, and the effects seem to vary markedly across people. Davis et al. (1950) reported three cases where exposures to intense tones for periods of 1–8 min produced permanent hearing loss. Exposures to tones with frequencies of 0.5, 2, and 4 kHz led to permanent hearing losses that had their maximal values at 3.4, 8, and 4 kHz, respectively. Thus, the relationship between the exposure frequency and the frequency at which the T-NIHL is greatest was highly variable.

The CLB method for diagnosing NIHL, and other similar methods, are based on the assumption that the maximum NIHL will occur for frequencies close to 4 kHz. Hence, these methods are entirely inappropriate in cases of T-NIHL produced by TSE. Coles et al. (2000) explicitly recognised this limitation in their paper, where they stated that the guidelines only apply to “typical” cases of NIHL produced by common types of broadband noise and that “Sounds not fitting this description include those predominantly of tonal nature.” The sounds produced by TSE are clearly of a tonal nature. These sounds cannot be classified as “broadband”, as their spectra are dominated by discrete sinusoidal components. Similarly, the method of Moore (2020) is not appropriate for diagnosing T-NIHL. Indeed, there are no generally accepted methods for diagnosing T-NIHL produced by the use of TSE. Here we make two recommendations for such methods.

For individuals who *exclusively* used only one ear when operating TSE, an appropriate method of diagnosing T-NIHL is to compare the audiograms for the exposed and non-exposed ears. If the mean audiometric threshold across 1, 2, 3, 4, 6 and 8 kHz is 5 dB or more higher for the exposed than for the non-exposed ear, then, in our view, this would indicate, on the balance of probability, that the exposure led to T-NIHL.

For individuals who used both ears with the TSE, the amount of T-NIHL cannot be safely assessed by comparing audiometric thresholds for the two ears. This is the case even when one ear was used only occasionally with the TSE, since only a small number of exposures may be sufficient to produce some T-NIHL. In cases where an individual used both ears with the TSE, a reasonable procedure is to compare the audiometric thresholds for each ear with the median audiometric thresholds for a person of the same age and gender with no known history of noise exposure, based on ISO 7029 (2017). If the mean audiometric threshold across 1, 2, 3, 4, 6, and 8 kHz is 5 dB or more higher than the median for a person of the same age and gender then, in our view, this would indicate, on the balance of probability, that the exposure led to T-NIHL.

## Quantification of NIHL

In this section we consider methods that can be used for quantifying NIHL, assuming that a positive diagnosis of NIHL has been reached using one of the methods described above.

### Exposure to Steady Broadband Noise

For individuals who have been exposed to steady broadband noise, two main methods are available for quantification. One is based on comparison of the measured HTLs with a reference database of HTLs for non-noise exposed individuals as a function of age, frequency and gender. Another method, which is widely used in the UK, is based on the guidelines of Lutman et al. (2016), referred to here as the LCB method. As with the CLB diagnostic method, the LCB quantification method was intended to be appropriate for the NIHL that occurs following long-term exposure to the type of broadband noise that typically occurs in factories. This is associated with a “notch” or a “bulge” in the audiogram, most commonly centred at 3, 4 or 6 kHz and with only a small threshold elevation at 8 kHz, unless the NIHL is severe (Passchier-Vermeer, 1974; Robinson, 1985; Smoorenburg, 1992). We consider first the LCB method and its limitations.

The LCB method involves two “passes”. Pass one is the same as for the CLB bulge analysis described above, using anchor points at 1 and 8 kHz. Pass two involves the steps illustrated in Table 3 using the same audiometric thresholds as for Table 1 and using the same AAHL values:

1. Estimation of the extent to which the audiometric thresholds at the anchor points include some NIHL, based largely on the data of Passchier-Vermeer (1974). The NIHL value at 1 kHz is calculated as 0.15 times the estimated NIHL at 4 kHz obtained in the first pass. The NIHL value at 8 kHz is calculated as 0.4 times the estimated NIHL at 4 kHz (line F in Table 3). Note that this makes the method unsuitable when there is no audiometric notch at 4 kHz, the greatest hearing loss instead occurring at 3 or 6 kHz.
2. Altering the measured HTLs to create modified HTLs at the anchor points, by subtracting the estimated NIHL values from the measured HTLs (line G).
3. Selecting AAHL values to give a good match to the modified HTLs at the anchor points (line H). In the example given, the AAHL values are the same as for the first pass (line C), but they could in principle be different, if a different percentile is chosen.
4. Calculating “misfit values” at the anchor points, which are the differences between the modified HTLs (Line G) and the AAHL values (line H), giving the values in line I.
5. Interpolation of the misfit values in line I on a logarithmic frequency scale to give misfit values at all frequencies (line J).
6. Calculation of modified AAHL values by adding the AAHL values in line H to the interpolated misfit values in line J, giving line K.
7. Setting the modified AAHL values in line K to 0 when they are negative (line L).
8. Setting the modified AAHL values in line L to the measured HTLs when the modified AAHL values are greater than the measured HTLs (line M).
9. Quantifying NIHL as the difference between the measured HTLs (line A) and the values in line M, giving line N.

**Table 3.** Example of an LCB Analysis Using the Same HTLs as for Table 1 and using the same nominal AAHL values.

			Frequency, kHz					
	Pass 1		1	2	3	4	6	8
A	Hearing threshold level (HTL), dB HL		15	10	15	40	40	40
B	HTL at selected anchor points		15					40
C	Selected age-associated hearing loss (AAHL)		12.1	18.8	25.8	34.7	39.4	46.2
	Misfit values (dB) = B–C at anchor points		2.9					−6.2
D	Interpolated misfit values (dB)		2.9	−0.1	−1.6	−3.2	−4.6	−6.2
	Adjusted AAHL = C + D		15.0	18.7	24.1	31.6	35	40
	Set AAHL to 0 when AAHL<0		15.0	18.7	24.1	31.6	35	40
E	Set AAHL to actual when AAHL>actual		15.0	10.0	15.0	31.6	34.8	40
	NIHL (dB) = A − E		0.0	0.0	0.0	8.4	5.2	0
	Pass 2							
F	Estimate NIHL at anchor points (dB)		1.3					3.4
G	Modified HTL at anchor points (dB HL) = A − F		13.7					36.6
H	Selected age-associated hearing loss (AAHL)		12.1	18.8	25.8	34.7	39.4	46.2
I	Misfit values (dB) at anchor points = G–H		1.7					−9.5
J	Interpolated misfit values (dB)		1.7	−2.0	−3.9	−5.8	−7.6	−9.5
K	Modified AAHL (dB) = H + J		13.7	16.7	21.8	28.9	31.8	36.6
L	Set AAHL to 0 when AAHL<0		13.7	16.7	21.8	28.9	31.8	36.6
M	Set AAHL to actual HTL when AAHL>actual		13.7	10.0	15.0	28.9	31.8	36.6
N	NIHL (dB) = A − M		1.3	0.0	0.0	11.1	8.2	3.4
	Mean NIHL at 1, 2 and 3 kHz, dB	0.4						
	Mean NIHL at 1, 2 and 4 kHz, dB	4.1						

The AAHL values here were calculated to one decimal place using the equations in ISO 1999 (2013) but with the modified baseline values for young people used by Coles et al. (2000). Values in specific lines are denoted A to N.

For the example shown in Table 3, the estimated NIHL is 0.4 dB when averaged across 1, 2, and 3 kHz, and 4.1 dB when averaged over 1, 2, and 4 kHz. Some problems with the LCB method are immediately apparent from this example. Recall that the percentile for the AAHL values was selected as the 25^th^ (worst) so as to give a reasonable match to the measured HTLs at 1 and 8 kHz. However, with this percentile, the measured HTLs at 2 and 3 kHz are markedly lower (better) than the selected AAHL values. This suggests that, in the absence of noise exposure, this individual would have fallen at a better percentile than the 25th. Furthermore, changing the selected percentile only changes the outcome of the LCB method slightly, because the AAHL values are adjusted to be close to the measured HTLs at the anchor points of 1 and 8 kHz. For example, if the 50^th^ percentile is selected, the estimated NIHL remains 0.4 dB when averaged across 1, 2, and 3 kHz, and changes to 3.9 dB when averaged over 1, 2, and 4 kHz. It appears very likely that the NIHL of this individual is under-estimated when the LCB method is used.

A widely used alternative is to quantify NIHL by comparing the measured HTLs with AAHL values, based on published standards such as ISO 7029 (2017) or on other normative data (Flamme et al., 2020). To do this, a default percentile can be used, such as 50%, or an appropriate percentile can be selected for the individual concerned. For the case illustrated in Table 3, a reasonable match to the HTLs at 1, 2, and 3 kHz is obtained using the 30^th^ (worst) percentile for a 50 year old man in ISO 7029 (2017). Table 4 illustrates the application of this method to the same case as for Table 3, using AAHL values for the 30^th^ percentile. The estimated NIHL is 2.5 dB averaged across 1, 2, and 3 kHz, and 9.9 dB averaged over 1, 2, and 4 kHz, values more than double those obtained with the LCB method. However, these values may still under-estimate the true NIHL of this individual, since it is likely that the noise exposure had some effect at 2 and 3 kHz, and that this individual would have had even better HTLs than those measured if the individual had not been noise exposed.

**Table 4.** Quantification of NIHL for the Same Case as in Tables 1–3, but based on comparison of the measured HTLs with AAHL values for a non-exposed man at the 30^th^ (worst) percentile based on ISO 7029 (2017).

Age, years	50						
Percentile	30						
Frequency, kHz		1	2	3	4	6	8
Hearing threshold level, dB HL		15	10	15	40	40	40
Age-associated hearing loss (AAHL), dB HL		7.6	11.6	14.9	17.9	22.5	26.0
Estimated noise-induced hearing loss (NIHL), dB		7.4	−1.6	0.1	22.1	17.5	14.0
Set NIHL to 0 if NIHL < 0		7.4	0.0	0.1	22.1	17.5	14.0
Mean M-NIHL at 1, 2 and 3 kHz, dB	**2.5**						
Mean M-NIHL at 1, 2 and 4 kHz, dB	**9.9**						

Table 5 shows an analysis for the same individual but assuming the 50^th^ percentile rather than the 30^th^ percentile. Now, the estimated NIHL values are even larger, reaching 6.9 dB averaged across 1, 2, and 3 kHz, and 14.6 dB averaged over 1, 2, and 4 kHz. Clearly, the choice of percentile has a large effect on the estimated NIHL. For this particular case, the NIHL values probably fall between the values shown in Table 4 and those shown in Table 5, since Table 4 represents a probable lower bound to the NIHL and Table 5 represents a probable upper bound.

**Table 5.** As Table 4 but with AAHL values for a non-exposed man at the 50^th^ (median) percentile, based on ISO 7029 (2017).

Age, years	50						
Percentile	50						
Frequency, kHz		1	2	3	4	6	8
Hearing threshold level, dB HL		15	10	15	40	40	40
Age-associated hearing loss (AAHL), dB HL		4.0	6.5	8.7	10.7	13.8	16.1
Estimated noise-induced hearing loss (NIHL), dB		11.0	3.5	6.3	29.3	26.2	23.9
Set NIHL to 0 if NIHL < 0		11.0	3.5	6.3	29.3	26.2	23.9
Mean M-NIHL at 1, 2 and 3 kHz, dB	**6.9**						
Mean M-NIHL at 1, 2 and 4 kHz, dB	**14.6**						

There is no single method for selecting an appropriate percentile that is always applicable. One method is by consideration of one or more audiograms obtained before the noise exposure occurred. This approach is based on the assumption that better hearing in early life is associated with a slower rate of decline of hearing with increasing age, consistent with ISO 7029 (2017). For example, Linssen et al. (2014) showed that for HTLs averaged across the frequencies 1, 2, and 4 kHz (denoted PTA) the rate of increase of PTA in dB/year was approximately linearly related to the PTA at the start of the measurement period. As a result, in the absence of noise exposure or ear pathology, an individual stays roughly at the same percentile throughout their life. A problem with this approach is that audiograms obtained many years ago are often of uncertain reliability, and many omit measurement of HTLs at 8 kHz. Hence, caution is advised in using such audiograms to select the appropriate percentile unless there is reason to believe that the early audiograms have been obtained under known suitable conditions according to a recognized standard method.

Another method is to select the percentile based on the HTLs for the frequencies with the best HTLs, for the better hearing ear. This method was used to select the percentile for the case illustrated in Table 4. However, this method has the disadvantage that it may lead to substantial under-estimation of the magnitude of NIHL when the NIHL has affected HTLs at most or even all frequencies.

In our opinion, the fairest approach is to assume the 50^th^ percentile by default unless there is good evidence that the hearing of the individual was unusually good or bad prior to the start of noise exposure. Some individuals may have had better pre-noise-exposure hearing than the median and some may have had worse hearing than the median, but the use of median values will give a fair quantification of NIHL in typical cases.

### Cases of Exposure to Impulsive Sounds in Industry

For cases of exposure to impulsive sounds in industrial settings, we recommend that quantification is based on the same method as described above, by comparing the measured HTLs with the AAHL values specified in ISO 7029 (2017). The 50^th^ percentile should be used unless there is good evidence that the hearing of the individual was unusually good or bad prior to the start of noise exposure.

### Exposure to Noise During Military Service

The LCB method is entirely inappropriate for quantifying M-NIHL, because it is based on the assumption that HTLs at 1 and 8 kHz have been only minimally affected by the noise exposure, and this is rarely the case for noise exposure during military service (Lowe & Moore, 2021; Moore, 2020). This is illustrated in Table 6, which shows the application of the LCB method to an example military veteran aged 47 years, taken from the data of Lowe and Moore (2021). The AAHL values were selected as those from Lutman et al. (2016) for a 47 year old man at the 5^th^ (worst) percentile, since this gave a reasonable match to the measured HTLs at the anchor points of 1 and 8 kHz. The estimated M-NIHL was very small, having a maximal value of 2.1 dB at 3 kHz.

**Table 6.** Application of the LCB Quantification Method to a 47 Year old Military Veteran Using AAHL Values for a 47-Year old man at the 5^th^ (Worst) Percentile Using Values from (Lutman et al., 2016).

Lutman et al. (2016) method					Frequency, kHz		
Pass 1		1.0	2	3	4	6	8
Hearing threshold level (HTL), dB HL		20	20	40	50	45	65
HTL at selected anchor points		20.0					65.0
Selected age-associated hearing loss (AAHL)		19.4	28.2	36.5	47.4	53.9	62.4
Misfit values (dB)		0.6					2.6
Interpolated misfit values (dB)		0.6	1.3	1.6	1.9	2.3	2.6
Adjusted AAHL		20.0	29.4	38.1	49.4	56.1	65.0
Set AAHL to 0 when AAHL<0		20.0	29.4	38.1	49.4	56.1	65.0
Set AAHL to actual when AAHL>actual		20.0	20.0	38.1	49.4	45.0	65.0
Bulge (dB)		0.0	0.0	1.9	0.6	0.0	0.0
Pass 2							
Modified HTL at anchor points (dB)		19.9					64.7
Selected age-associated hearing loss (AAHL)		19.4	28.2	36.5	47.4	53.9	62.4
Misfit values (dB)		0.5					2.3
Interpolated misfit values (dB)		0.5	1.1	1.4	1.7	2.0	2.3
Modified AAHL (dB)		19.9	29.3	37.9	49.2	55.9	64.7
Set AAHL to 0 when AAHL<0		19.9	29.3	37.9	49.2	55.9	64.7
Set AAHL to actual when AAHL>actual		19.9	20.0	37.9	49.2	45.0	64.7
Modified bulge = noise-induced loss (dB)		0.1	0.0	2.1	0.8	0.0	0.3
Mean noise-induced loss 1, 2 and 3 kHz	0.7						
Mean noise-induced loss 1, 2 and 4 kHz	0.3						

The authors of the LCB method partly recognized this problem and stated that “cases will arise where the threshold at 8 kHz is clearly out of line with the trend for age-associated hearing loss and an alternative approach is required. In such circumstances, it is recommended that the user of the Guidelines should select a threshold value at 8 kHz that is in line with the overall trend for age-associated hearing loss, instead of the measured value, to use in the calculations” (Lutman et al., 2016, page 357). Table 7 illustrates the effect of adjusting the HTL at 8 kHz to be 45 dB HL, corresponding to the 20^th^ (worst) percentile for a man aged 47 years, and using the corresponding AAHL values in the LCB calculations. The AAHL value at 2 kHz for this percentile (19 dB HL) is close to the measured HTL of 20 dB HL at 2 kHz. Now the estimated M-NIHL is markedly larger, reaching about 18 dB at 4 kHz. The mean across 1, 2, and 3 kHz is 5.5 dB and the mean across 1, 2, and 4 kHz is 6.6 dB.

**Table 7.** As Table 6, but with the HTL at 8 kHz adjusted to 45 dB HL and with the percentile changed to the 20^th^ (worst).

Lutman et al. (2016) method					Frequency, kHz		
Pass 1		1.0	2	3	4	6	8
Hearing threshold level (HTL), dB HL		20	20	40	50	45	45
HTL at selected anchor points		20.0					45.0
Selected age-associated hearing loss (AAHL)		12.5	19.0	25.3	33.8	38.4	44.6
Misfit values (dB)		7.5					0.4
Interpolated misfit values (dB)		7.5	5.1	3.9	2.7	1.6	0.4
Adjusted AAHL		20.0	24.1	29.3	36.5	40.0	45.0
Set AAHL to 0 when AAHL<0		20.0	24.1	29.3	36.5	40.0	45.0
Set AAHL to actual when AAHL>actual		20.0	20.0	29.3	36.5	40.0	45.0
Bulge (dB)		0.0	0.0	10.7	13.5	5.0	0.0
Pass 2							
Modified HTL at anchor points (dB)		18.0					39.6
Selected age-associated hearing loss (AAHL)		12.5	19.0	25.3	33.8	38.4	44.6
Misfit values (dB)		5.4					−5.0
Interpolated misfit values (dB)		5.4	2.0	0.2	−1.6	−3.3	−5.0
Modified AAHL (dB)		18.0	21.0	25.5	32.2	35.1	39.6
Set AAHL to 0 when AAHL<0		18.0	21.0	25.5	32.2	35.1	39.6
Set AAHL to actual when AAHL>actual		18.0	20.0	25.5	32.2	35.1	39.6
Modified bulge = noise-induced loss (dB)		2.0	0.0	14.5	17.8	9.9	5.4
Mean noise-induced loss 1, 2 and 3 kHz	5.5						
Mean noise-induced loss 1, 2 and 4 kHz	6.6						

In practice, the selection of an appropriate adjusted HTL at 8 kHz (or at 1 kHz) is not straightforward, and different “experts” may select different adjusted HTLs, leading the method to be open to manipulation. Furthermore, even quite small adjustments to the HTLs at 1 and 8 kHz can have a substantial effect. For example, adjusting the HTL at 1 kHz from 20 to 10 dB HL (leaving the adjusted HTL at 8 kHz at 45 dB HL) almost doubles the estimated M-NIHL averaged across 1, 2, and 3 kHz, from 5.5 to 9.8 dB.

In our opinion, the most appropriate method for quantifying M-NIHL is by comparison with the HTLs expected from the 50^th^ percentile of ISO 7029 (2017), as described above. Table 8 illustrates the results obtained with this method for the same case as in Tables 6 and 7. The estimated M-NIHL is markedly larger using this method, reaching 41.5 dB at 4 kHz. The mean across 1, 2, and 3 kHz is 21.6 dB and the mean across 1, 2, and 4 kHz is 24.4 dB.

**Table 8.** Estimation of the Amount of M-NIHL by Comparison to the 50^th^ Percentile of ISO 7029 (2017) for the Same Case as in Tables 6 and 7.

Age, years	47						
Percentile	50						
Frequency, kHz		1	2	3	4	6	8
Hearing threshold level, dB HL		20	20	40	50	45	65
Age-associated hearing loss (AAHL), dB HL		3.1	5.1	6.9	8.5	11.0	12.8
Estimated noise-induced hearing loss (NIHL), dB		16.9	14.9	33.1	41.5	34.0	52.2
Set NIHL to 0 if NIHL < 0		16.9	14.9	33.1	41.5	34.0	52.2
Mean M-NIHL at 1, 2 and 3 kHz, dB	**21.6**						
Mean M-NIHL at 1, 2 and 4 kHz, dB	**24.4**						

In some cases, it may be appropriate to use a percentile other than the 50^th^. Reasons for doing this are:

1. There are one or more reliable audiograms obtained prior to the start of noise exposure that indicate markedly worse or better hearing than average. If so, the percentile should be based on the pre-exposure audiogram(s).
2. A recent audiogram shows HTLs at one or more frequencies that indicate hearing better than the 50^th^ percentile for that individual's age. For example, if a 47 year old man shows an HTL at 8 kHz of 10 dB HL, corresponding to the 65^th^ percentile in ISO 7029 (2017), then it would be appropriate to use the 65^th^ percentile.
3. If one ear has markedly better hearing than the other ear, it is appropriate to base the choice of percentile on the HTLs for the better-hearing ear.

The use of a higher (better) percentile will increase the estimated M-NIHL, while the use of a lower (worse) percentile will decrease the estimated M-NIHL, as illustrated in Table 9, which shows the same analysis as for Table 8, but with the percentile changed from the 50^th^ to the 25^th^. In this case, the mean estimated M-NIHL across 1, 2, and 3 kHz is reduced to 16.1 dB and the mean across 1, 2, and 4 kHz is reduced to 18.5 dB. However, even these reduced values are markedly greater than the values obtained using the LCB method using the unadjusted HTLs (Table 6) and with the HTL at 8 kHz adjusted (Table 7).

**Table 9.** As Table 8, but using the 25^th^ (worst) percentile from ISO 7029 (2017), instead of the 50^th^ percentile.

Age, years	47						
Percentile	25						
Frequency, kHz		1	2	3	4	6	8
Hearing threshold level, dB HL		20	20	40	50	45	65
Age-associated hearing loss (AAHL), dB HL		7.1	10.8	13.9	16.6	20.8	24.1
Estimated noise-induced hearing loss (NIHL), dB		12.9	9.2	26.1	33.4	24.2	40.9
Set NIHL to 0 if NIHL < 0		12.9	9.2	26.1	33.4	24.2	40.9
Mean M-NIHL at 1, 2 and 3 kHz, dB	**16.1**						
Mean M-NIHL at 1, 2 and 4 kHz, dB	**18.5**						

In summary, M-NIHL, like NIHL associated with exposure to steady broadband sounds, should be quantified by comparison to AAHL values taken from ISO 7029 (2017), using the 50^th^ percentile unless there are good reasons to choose a different percentile.

### Exposure to Intense Tones

As for M-NIHL, quantification using the LCB method is entirely inappropriate in cases of T-NIHL, for the same reasons as given in the discussion of the diagnosis of T-NIHL. Hence, once again, we recommend that quantification is based on comparison to AAHL values taken from ISO 7029 (2017), using the 50^th^ percentile unless there are good reasons to choose a different percentile.

### Choice of Reference Database

We recommend that NIHL should be quantified by comparison to ISO 7029 (2017), since the populations used to develop ISO 7029 (2017) were carefully screened to exclude both conductive hearing loss and noise exposure. However, it might be argued that a less carefully screened population should be used for comparison. One candidate database is that published by Flamme et al. (2020), which is based on a sample of 9937 individuals tested as part of the U.S. National Health and Nutrition Examination Survey (NHANES). The NHANES data are representative of the non-institutionalised, non-military U.S. population. Flamme et al. (2020) stated that “Cross-sectional trends are influenced by the combined effects of events (e.g. acute disorders, trauma, infection) and conditions that might be rare on the individual level (e.g. hereditary/genetic disorders) but have a collective impact on the distribution of hearing thresholds at the population level. These effects would be increasingly potent as a function of increased time at risk (i.e. correlated with age, but not an inexorable effect of age). The effects would be minimal on the tail of the distribution with better hearing sensitivity and would increase as consideration moves to the opposite tail of the distribution.” For these reasons, Flamme et al. (2020) recommended the use of the 75^th^ (best) percentile for estimating AAHL values and for estimating longitudinal trends. Flamme et al. (2020) found that AAHL values for frequencies from 3 to 8 kHz were slightly better for non-hispanic black (NHB) people than for the remainder of the population.

**Figure 1.**
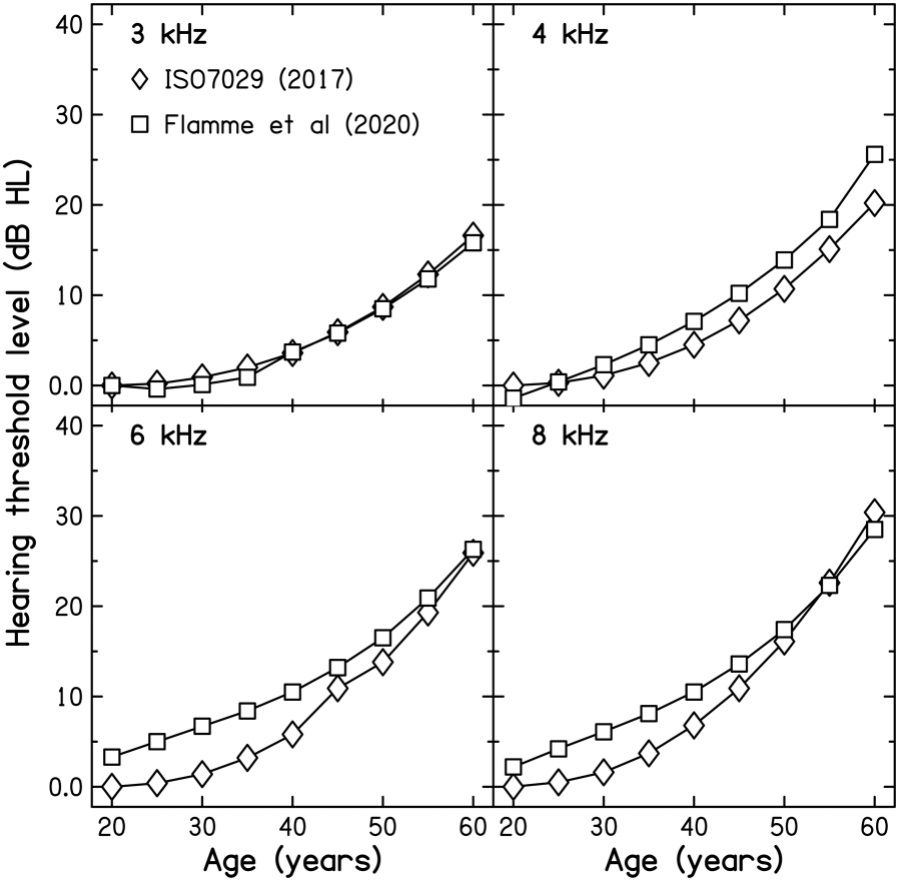
AAHL values for men expected from the 50^th^ percentile of ISO 7029 (2017) (diamonds) and from the values published by Flamme et al. (2020) (squares), plotted as a function of age. Each panel shows results for one frequency.

It turns out that, for ages up to 60 years, the AAHL values for the population evaluated by Flamme et al. (2020), excluding NHB people, are very close to those for the 50^th^ percentile of ISO 7029 (2017) for frequencies from 1 to 3 kHz, and differ only slightly for higher frequencies, as illustrated in Figure 1. For NHB individuals the AAHL values of Flamme et al. (2020) are even closer to those for the 50^th^ percentile of ISO 7029 (2017). Hence, the choice of reference database has little effect on the estimated amount of NIHL, especially when averaged across 1, 2, and 3 kHz, or 1, 2, and 4 kHz.

## The Use of Multiple Audiograms

It often happens that there are multiple post-exposure audiograms available for a given individual. If there are two or more audiograms obtained within a reasonably short period of time, say one or two years, we recommend averaging the HTLs across all of those audiograms to reduce measurement errors, unless there are good reasons for excluding one or more of the audiograms. Valid reasons for exclusion of a specific audiogram are:

1. Evidence that the audiogram was not obtained according to a recognized standard method, such as that recommended by the British Society of Audiology (2018).
2. When the HTLs are markedly worse than for two or more other audiograms, especially at low frequencies, which might indicate a collapsed ear canal or a temporary conductive loss, caused, for example, by congestion following a cold.

It can also happen that audiograms are available over a wide time period, from close to the end of noise exposure to many years after the end of the exposure. In such cases, the question arises as to which of the available audiograms most accurately reflects the effects of the noise exposure. It is traditionally believed that the effects of exposure to noise cease once the exposure itself has ceased (Humes et al., 2006; Mirza et al., 2018). If this is the case, exposure to noise should not affect the progression of hearing loss with increasing age after the exposure ceases, and estimates of the amount of NIHL should not be affected by whether the audiogram was obtained soon after or long after the noise exposure ceased. However, the data on which this traditional belief is based were largely obtained from populations of older people (aged 70 years or more), and even the non-noise exposed participants had substantial hearing loss at high frequencies (Hederstierna & Rosenhall, 2016; Lee et al., 2005). Thus, it is not clear from these data whether the progression of hearing loss after the end of noise exposure is affected for younger people with small or no hearing loss at the end of the exposure.

Studies using mice indicate that noise exposure can accelerate the progression of hearing loss following the exposure, when there is little or no hearing loss immediately after the exposure (Fernandez et al., 2015; Kujawa & Liberman, 2006). Kujawa and Liberman (2006) concluded that “Data suggest that pathologic but sublethal changes initiated by early noise exposure render the inner ears significantly more vulnerable to aging.” Data from humans exposed to noise during military service support this idea (Kim et al., 2017; Moore, 2021; Xiong et al., 2014).

Moore (2021) argued that mild to moderate hearing loss is usually primarily a consequence of loss of function of the outer hair cells (OHCs) in the cochlea (Borg et al., 1995). Some damage to the OHCs can occur with little or no change in the threshold for detecting sounds (Dallos & Harris, 1978; Evans & Harrison, 1976; Glavin et al., 2021; Harrison & Evans, 1979), consistent with the concept of a “cochlear reserve”; the cochlea can sustain some damage without loss of function as revealed by the audiogram. However, once the reserve is sufficiently depleted, effects in the audiogram become apparent with further damage, as can occur with increasing age. Hearing loss up to 55 dB following a period of noise exposure could be due primarily to loss of OHC function. In this case, acceleration of the subsequent progression of hearing loss due to further OHC damage is not expected. However, if the hearing loss at the end of noise exposure is much less than 55 dB at some frequencies, then there is scope for acceleration of the subsequent progression of hearing loss at those frequencies due to further damage to OHCs. This led Moore (2021) to propose the following hypothesis: for frequencies where the NIHL at the end of noise exposure is mild, the subsequent progression of hearing loss is accelerated. In contrast, for frequencies where the NIHL is moderate or severe at the end of the noise exposure, the subsequent progression of hearing loss is unaffected or is slowed. The hypothesis was proposed specifically in relation to M-NIHL, but it might apply to other forms of NIHL.

Moore and Lowe (2022) tested this hypothesis using longitudinal data obtained from 29 former male military personnel. Audiograms obtained close to the end of military service were compared with those obtained five or more years later. Rates of change of HTL in dB/year were compared with those expected from ISO 7029 (2017) for men at the 50^th^ percentile. The results showed that the progression of hearing loss following the end of military service was accelerated for frequencies where the hearing loss was absent or mild at the end of military service, by about 1.7 dB/year on average for frequencies from 3 to 8 kHz, but the progression was unaffected or slowed for frequencies where the hearing loss at the end of military service exceeded about 50 dB. Acceleration, when present, occurred over a wide frequency range, including 1 kHz.

It is not yet clear whether similar effects are produced by exposure to noises other than those encountered during military service, for example at rock concerts or from work in heavy industries. However, the studies showing acceleration of the progression of hearing loss following noise exposure in mice suggest that similar effects will occur, since these studies used steady noise as the exposure stimulus, rather than impulsive sounds (Fernandez et al., 2015; Kujawa & Liberman, 2006). It is also known that noise exposure of all types can result in tinnitus that sometimes starts many years after the noise exposure has ceased (Axelsson & Barrenas, 1992; Henry et al., 2010), supporting the idea that some effects of noise exposure are only revealed when further deterioration to the auditory system occurs as a result of aging and other factors.

Given the evidence supporting the hypothesis that noise exposure during military service can affect the subsequent progression of hearing loss with increasing age, we recommend that when audiograms are available both close to the end of military service and some time afterwards, the most recent audiograms are used to diagnose and quantify M-NIHL, since the most recent audiograms include any effects of the noise exposure on the current hearing loss. However, this is problematic when there has been significant noise exposure from work or leisure activities following the end of military service. Where there has been such exposure, then audiogram(s) obtained soon after the end of military service may be of greater relevance.

It may also be appropriate to use the most recent audiograms when diagnosing and quantifying NIHL caused by non-military exposures, but more evidence is required to assess this.

## Frequencies to be Used When Quantifying NIHL

In medico-legal cases, compensation is often based on an average of the NIHL across certain frequencies for each ear. In some countries, compensation for occupational NIHL has traditionally been based on the mean estimated NIHL at 1, 2 and 3 kHz (UK, King et al., 1992) or 0.5, 1, 2 and 3 kHz (USA, American Medical Association, 2008; Dobie, 2011). However, there are strong arguments for including 4 kHz in the overall estimate of NIHL (Moore, 2016, 2020) and in some countries, such as Ireland and Australia, 4 kHz is included.

Hearing aids can be quite effective at improving the ability to understand speech in quiet, but they are less effective at improving the ability to understand speech in noise (Plomp, 1978; Souza, 2016). The primary complaint of people with hearing loss is difficulty in understanding speech when background sounds are present (Moore, 2007). Therefore, the most appropriate audiometric frequencies to take into account when assessing hearing disability are those that give the most accurate prediction of the ability to understand speech in noise.

Kryter et al. (1962) studied the relationship between scores on a variety of speech tests and the characteristics of the audiogram, for participants with a wide range of audiometric configurations. They stated that “the ability to perceive speech can be predicted as well by the hearing thresholds at 2000, 3000, and 4000 cps alone as it can by including the losses at all the other frequencies tested” and concluded that “the three most important test frequencies to use for predicting the ability to understand speech would be 2000, 3000, and 4000 cps.”

Smoorenburg (1992) studied the effects of NIHL produced by exposure to steady noise in factories on the ability to understand speech in noise and of the relationship of that ability to the audiogram. He measured the speech reception threshold (SRT) at which 50% of sentences in noise could be understood. The best two-frequency predictor of the SRT was the average of the HTLs at 2 and 4 kHz. Smoorenburg also examined which single HTL gave the most accurate prediction of the SRT. He found that the HTL at 4 kHz gave the most accurate prediction, although HTLs at 3 and 6 kHz gave predictions that were nearly as accurate. These results clearly indicate that the hearing loss at high frequencies (2–6 kHz) is the best predictor of the intelligibility of speech in noise for people with NIHL.

Wilson (2011) tested 3266 military veterans, many of whom had been exposed to intense noise including impulsive sounds. The intelligibility of speech in noise was assessed using the Words-in-Noise (WIN) test, which assesses word recognition in multi-talker babble at seven signal-to-noise ratios (SNRs) and uses the 50% correct point (in dB SNR) as the primary outcome measure. Scores on the WIN were predicted significantly better by the average HTL at 1, 2 and 4 kHz than by the average HTL at 0.5, 1, and 2 kHz, confirming the importance of high-frequency hearing for the ability to understand speech in noise.

Overall, it is very clear that any assessment of the overall magnitude of NIHL should include the HTL at 4 kHz. We recommend that the average HTL across 1, 2, and 4 kHz is used to quantify the overall magnitude of NIHL for a given ear.

## Summary of Recommendations

1. When assessing claims for compensation for occupational NIHL, a comprehensive medical examination should be conducted to assess possible causes of hearing loss other than noise exposure, to assess the exposure history of the individual, and to assess tinnitus and hyperacusis, preferably using validated measures.
2. It should be established that noise exposure sufficient to produce hearing loss in at least 10% of individuals has occurred. For people who have been exposed primarily to steady broadband noise, a total noise exposure of 90 dB(A) NIL is sufficient. For people who have regularly been exposed to impulsive sounds in non-military occupations, a lower limit of 86 dB(A) NIL should be used. All those who have seen active military service or who have worked with TSE are likely to have been exposed to sounds with the potential to cause hearing loss.
3. For people who have been exposed to steady broadband noise, an appropriate method of diagnosing NIHL is based on a modified version of the CLB method, using the following requirements:
R1(mod). A single measurement of the HTL at 3, 4 or 6 kHz should be at least 10 dB greater than the HTL at 1 kHz or 2 kHz.
R2(mod). There should be evidence for an NIL of 90 dB(A) or more.
R3(mod). There should be a downward notch or bulge in the audiogram in the range 3–6 kHz. A notch is defined as present when the HTL at 3 and/or 4 and/or 6 kHz is at least 10 dB greater than at 1 and 8 kHz. A bulge is defined as present when the HTL at 3 and/or 4 and/or 6 kHz is at least 10 dB greater than expected from AAHL values. To establish whether R3(mod) is satisfied, a bulge analysis using the HTLs at 1 kHz and at 8 kHz as “anchor points” should be conducted. The AAHL values should be based on ISO 7029 (2017). The percentile should be chosen to minimize the mismatch between the measured HTLs and the AAHL values at the anchor points of 1 and 8 kHz.

No adjustment should be made to allow for the use of THD39 headphones. The lower anchor frequency should be changed to 2 kHz when the HTL at 2 kHz is 10 dB or more better than the HTL at 1 kHz and the upper anchor frequency should be changed to 6 kHz when the HTL at 6 kHz is 10 dB or more better than the HTL at 8 kHz.

This method can also be used for people who have been exposed to impulsive sounds like hammering while working in heavy industry, but in that case R2(mod) is: There should be evidence for an NIL of 86 dB(A) or more.

4. For people who have been exposed to noise during military service, the diagnostic method of Moore (2020) is recommended. With this method, R0 and R1 M must be met and either R2aM or R2bM or both must be met. The requirements are:
  R0. Sufficient noise exposure has occurred. This is almost certainly the case for those who have seen active military service.
  R1 M. A single value of the HTL at 3, 4, 6, or 8 kHz is at least 10 dB higher than the HTL at 1 or 2 kHz.
  R2aM. The difference between HTLs at 8 and 6 kHz is at least 5 dB smaller than would be expected from age alone or the difference between HTLs at 8 and 4 kHz or between 8 and 3 kHz is at least 10 dB smaller than would be expected from age alone, based on the median values in ISO 7029 (2017).
  R2bM. The HTL at any one of 4, 6, or 8 kHz is at least 20 dB higher than the median threshold for each frequency expected for that age, based on ISO 7029 (2017).

For this method, confidence in the diagnosis is greatest when R1M, R2aM and R2bM are all met. Confidence is somewhat lower, but still high, when only R1M and R2aM are met. Confidence is lower (but still with a probability greater than 50%) when only R1M and R2bM are met. This method can also be applied to people who have been exposed to steady broadband noise or impulsive noise in factories when the NIL is 100 dB(A) or more.

5. For people who have been exposed to intense tones produced by TSE, T-NIHL can be diagnosed using one of two methods:
  - For individuals who *exclusively* used only one ear when operating TSE, if the mean audiometric threshold at 1, 2, 3, 4, 6 and 8 kHz is 5 dB or more higher for the exposed than for the non-exposed ear, this indicates T-NIHL.
  - For individuals who used both ears with the TSE, if the mean audiometric threshold at 1, 2, 3, 4, 6, and 8 kHz is 5 dB or more higher than the median for a person of the same age and gender as determined from ISO 7029 (2017), this indicates T-NIHL.
6. NIHL of all types can be quantified by comparing the measured HTLs with the HTLs expected from age alone, based on ISO 7029 (2017) or on other normative data (Flamme et al., 2020). When ISO 7029 (2017) is used, the AAHL values corresponding to the 50^th^ percentile should be selected unless there are good reasons to choose a different percentile.
7. If there are two or more audiograms obtained within a period of one or two years, the HTLs should be averaged across all of those audiograms to reduce measurement errors, unless there are good reasons for excluding one or more of the audiograms.
8. When audiograms are available both close to the end of military service and some time afterwards, provided that there has not been significant noise exposure after the end of military service the most recent audiograms should be used to diagnose and quantify M-NIHL, since these include any effects of the noise exposure on the current hearing loss. When there has been significant noise exposure after the end of military service, it may be more appropriate the use the audiograms obtained close to the end of military service. Alternatively, the most recent audiograms can be used, but the possible effects of the post-service noise exposure should be taken into account.
9. The overall extent of the NIHL for a given ear should be quantified as the average of the estimated NIHL values at 1, 2, and 4 kHz.
